# Altered subcortical emotional salience processing differentiates Parkinson’s patients with and without psychotic symptoms

**DOI:** 10.1016/j.nicl.2020.102277

**Published:** 2020-05-30

**Authors:** F. Knolle, S. Garofalo, R. Viviani, A. Justicia, A.O. Ermakova, H. Blank, G.B. Williams, G. Arrondo, P. Ramachandra, C. Tudor-Sfetea, N. Bunzeck, E. Duezel, T.W. Robbins, R.A. Barker, G.K. Murray

**Affiliations:** a Department of Psychiatry, University of Cambridge, Cambridge, UK; b Department of Neuroradiology, Technical University Munich, Munich, Germany; c University of Bologna, Department of Psychology, Bologna, Italy; d Institute of Psychology, University of Innsbruck, Innsbruck, Austria; e Psychiatry and Psychotherapy Clinic III, University of Ulm, Ulm, Germany; f IMIM (Hospital del Mar Medical Research Institute), Centro de Investigación Biomédica en Red de Salud Mental (CIBERSAM), Barcelona, Spain; g Faculty of Natural Sciences, Imperial College London, UK; h Institute of Systems Neuroscience, University Medical Center Hamburg-Eppendorf, Hamburg, Germany; i Department of Clinical Neuroscience and WT-MRC Cambridge Stem Cell Institute, University of Cambridge, Cambridge, UK; j Cambridgeshire and Peterborough NHS Foundation Trust, Cambridge, UK; k Institute of Psychology I, University of Lübeck, Lübeck, Germany; l Otto-von-Guericke University Magdeburg, Institute of Cognitive Neurology and Dementia Research, Magdeburg, Germany; m German Centre for Neurodegenerative Diseases (DZNE), Magdeburg, Germany; n Department of Psychology, University of Cambridge, Cambridge, UK

## Abstract

**Objective:**

Current research does not provide a clear explanation for why some patients with Parkinson’s Disease (PD) develop psychotic symptoms. The ‘aberrant salience hypothesis’ of psychosis has been influential and proposes that dopaminergic dysregulation leads to inappropriate attribution of salience to irrelevant/non-informative stimuli, facilitating the formation of hallucinations and delusions. The aim of this study is to investigate whether non-motivational salience is altered in PD patients and possibly linked to the development of psychotic symptoms.

**Methods:**

We investigated salience processing in 14 PD patients with psychotic symptoms, 23 PD patients without psychotic symptoms and 19 healthy controls. All patients were on dopaminergic medication for their PD. We examined emotional salience using a visual oddball fMRI paradigm that has been used to investigate early stages of schizophrenia spectrum psychosis, controlling for resting cerebral blood flow as assessed with arterial spin labelling fMRI.

**Results:**

We found significant differences between patient groups in brain responses to emotional salience. PD patients with psychotic symptoms had enhanced brain responses in the striatum, dopaminergic midbrain, hippocampus and amygdala compared to patients without psychotic symptoms. PD patients with psychotic symptoms showed significant correlations between the levels of dopaminergic drugs they were taking and BOLD signalling, as well as psychotic symptom scores.

**Conclusion:**

Our study suggests that enhanced signalling in the striatum, dopaminergic midbrain, the hippocampus and amygdala is associated with the development of psychotic symptoms in PD, in line with that proposed in the ‘aberrant salience hypothesis’ of psychosis in schizophrenia.

## Highlights

• Emotional salience processing differentiates PD patients with and without psychosis.
• Enhanced striatal, hippocampal and midbrain responses in PD patients with psychosis.
• Indication for ‘jumping to conclusions’ bias in the same PD patients with psychosis.
• Aberrant top-down and salience processing associated with PD psychosis.
• Similar deficits as proposed in ‘aberrant salience hypothesis’ of schizophrenia.

## 1 Introduction

Parkinson’s disease (PD) patients frequently suffer from psychotic symptoms which most commonly takes the form of visual hallucinations, delusions and illusions (Aarsland et al., 1999). With disease progression psychotic symptoms may shift to other modalities such as the auditory domain, comprising auditory hallucinations of incomprehensible voices (Inzelberg et al., 1998) or non-verbal sounds (Fenelon, 2000). PD psychosis characterises a spectrum of such psychotic symptoms that occur throughout the course of the disease, but especially in those with longer disease duration, higher age and possibly higher doses of, or certain kinds of, dopaminergic medication, giving an overall prevalence of 26% (Forsaa et al., 2010, Gibson et al., 2013, MacK et al., 2012). Subsequently, risk and modulatory factors include genetics, the use of dopamine-based antiparkinsonian drugs, and disease-specific factors such as cognitive impairment, dementia, duration and severity of PD, depression, sleep disorders, along with age and the presence of intercurrent infections or illnesses (Fénelon and Alves, 2010, Friedman, 2010, Morgante et al., 2012). Its development is associated with increased risk for mortality and nursing home placement as well as lower overall global functioning and well-being (Ffytche et al., 2017).

Current research suggests that PD psychosis combines a set of symptoms with a specific pathophysiology (comprehensive review (Ffytche et al., 2017)), rather than a single mechanistic dysfunction. Although there are clear differences between the primary psychiatric disorder of (schizophrenia spectrum) psychosis and PD psychosis, a disturbed dopaminergic system is a unifying element in both diseases, possibly contributing to the occurrence of psychotic symptoms in both disorders (Carter and Ffytche, 2015, Garofalo et al., 2017). PD psychosis is particularly interesting as it is commonly found as a comorbidity in PD patients but does not affect all.

A dysfunctional dopaminergic signal, perhaps in the mesolimbic regions, is argued to be associated with the inappropriate attribution of salience to otherwise irrelevant or non-informative stimuli, allowing for the formation of hallucinations and delusions; this theory has been termed the ‘aberrant salience hypothesis’ of psychosis (Heinz, 2002, Kapur, 2003, Roiser et al., 2013). Some models propose that within the hippocampal-striatal-midbrain circuits, hippocampal dysfunction leads to an enhanced subcortical dopaminergic signalling through descending projections to the ventral striatum (Lisman and Grace, 2005, Lodge and Grace, 2007). Supporting the involvement of these circuits, a recent study investigating novelty salience processing reported increased connectivity of hippocampal to striatal and midbrain regions, but decreased connectivity between the striatum and the midbrain in subjects at high risk of developing psychosis (Modinos et al., 2019). Furthermore, our previous work in first-episode psychosis patients, using the same salience paradigm as Modinos et al (2019), showed reduced midbrain, striatal and occipital activation while processing novelty and negative emotional salient stimuli (Knolle et al., 2018). In Parkinson’s disease research, a previous study showed that the use of a dopamine agonist (pramipexole or ropinirole) in young, medication-naïve PD patients led to an increase in aberrant motivational salience by facilitating arbitrary and illusory associations between stimuli and rewards with faster reaction times to task-irrelevant stimuli (Nagy et al., 2012). Unmedicated patients in that latter study did not suffer from psychotic symptoms, but had increased subscales on the O-LIFE unusual experience score after treatment with dopaminergic agents (Nagy et al., 2012). The authors suggest that the dopamine-agonist therapy to treat Parkinsonian symptoms might give rise to psychotic symptoms as the disorder progresses. Furthermore, another study (Mannan et al., 2008) suggested impaired salience processing in PD: in an eye-gaze experiment, patients showed an impaired ability to detect a salient stimulus in a visual search task. No psychotic symptoms were reported for the patients in that latter study. In our previous work (Garofalo et al., 2017), we investigated reward processing, a form of motivation salience, in PD patients with and without psychotic symptoms and in controls. PD patients with psychotic symptoms showed very similar patterns of reduced activation (including in the striatum and cingulate cortex) as reported in primary psychosis individuals (Ermakova et al., 2018, Murray et al., 2008).

In the current study, we sought to explore whether the ‘aberrant salience hypothesis’ of psychosis can be applied to psychosis seen in PD, affecting not only reward based salience (Garofalo et al., 2017), but also non-motivational salience. By so doing we sought to provide an explanation as to how psychotic symptoms arise in PD patients (Poletti, 2018). To our knowledge, the current study is the first to investigate brain responses to non-motivational salient visual stimuli in patients with and without PD psychotic symptoms. The comparison between the two patient groups is our main focus in this study. Additionally, we tested healthy controls. We used an fMRI salience paradigm (Bunzeck and Düzel, 2006) that previously has shown significantly altered midbrain, striatal, hippocampal and amygdala activations and connectivity in early stages of “psychiatric” psychosis in young adults (Knolle et al., 2018, Modinos et al., 2019).

This salience paradigm designed by Bunzeck and Düzel (2006) provides a multidimensional approach to salience, with four types of salient oddballs. As outlined in the orignial study four different types of salience (i.e., novelty, emotional salience, rareness and targetness) can be investigated. Rareness, however, is a frequency oddball generated using the contrast of the neutral oddball minus neutral standard, these two stimuli only vary in frequency but not in content. The other salience types (i.e., negative emotional salience, novelty, targetness) are differ in content and are matched in frequency, due to being generated from a contrast with the neutral oddball. (i.e., novelty – neutral, emotional – neutral, targetness – neutral). In this study, we concentrated on negative emotional salience for two reasons. First, PD shows a progressive and chronic degeneration of the nigrostriatal and mesocorticolimbic dopaminergic systems (Braak et al., 2005, Wu et al., 2012) and an impaired dopaminergic pathway of emotional processing (Badgaiyan, 2010, Laviolette, 2007, Salgado-Pineda et al., 2005). PD patients therefore show a wide range of emotional processing deficits (see recent reviews: (Moonen et al., 2017, Péron et al., 2012)), mainly linked to n abnormalities in predominantly ventral regions of the affective neurocircuitry. Second, in our recent study using the same paradigm in patients with an early psychosis, we found the strongest and most robust effect to be in emotional salience (Knolle et al., 2018). Based on the literature of deficits in emotional processing in PD and our previous findings, we hypothesised, first, that PD psychosis patients would demonstrate altered negative emotional salience brain responses in the ventral dopaminergic midbrain (i.e. substantia nigra/VTA), striatum, hippocampus and amygdala compared to healthy controls and, second, that PD patients without psychotic symptoms would show intermediate processing in response to negative emotional salience compared to healthy controls and PD patients with psychotic symptoms.

The aberrant salience theory of psychosis has posited that whilst perceptual salience may be misattributed in psychosis, higher-order cognitive processes are invoked to shape abnormal experiences into abnormal beliefs. Whilst our focus in the current study is on brain correlates of emotional salience processing, in a preliminary analysis we also examined whether higher-order (probabilistic) reasoning is affected in PD psychosis.

## 2 Methods

### 2.1 Subjects

In total, we recruited 26 participants, who had a diagnosis of PD without any psychotic symptoms using established diagnostic criteria; 15 participants with a diagnosis of PD and ongoing or previous psychotic symptoms, and 19 healthy control subjects, without any history of neurological or psychiatric disorder, matched for age, gender and education (see Table 1). We assessed psychotic symptoms in Parkinson’s patients using the Comprehensive Assessment of At Risk Mental States (CAARMS) (Yung et al., 2005) as well as the Positive and Negative Symptom Scale (PANSS) (Kay et al., 1987) – also see our previous work (Garofalo et al., 2017) for a detailed description) and Table 1. For two participants, one healthy control and one PD patient with psychotic symptoms, the fMRI session had to be aborted during scanning of the relevant task, as both participants felt uncomfortable inside the scanner. Both participants decided not to continue with the scanning and so were excluded from any analysis. Additionally, two PD patients without psychotic symptoms were excluded due to excessive movement artefact in the scanner (see details below). Finally, two outliers were identified during our analysis, one healthy control and one PD patient without psychotic symptoms, who exceeded -/+ two standard deviations from the averaged imaging signal in all regions of interest (ROI). The final sample therefore comprised of 52 participants: 23 PD patients without psychotic symptoms, 17 healthy controls and 14 PD patients with psychotic symptoms.

**Table 1:** Demographics and pathology of psychiatric aspects of Parkinson’s Disease.

Characteristics	Parkinson Control	Parkinson Psychosis*	Healthy Volunteers
*Demographics*			
Participants, n	23	14	17
Age, mean (SD) yr	63.1 (9.4)	62.5 (7.4)	63.1 (SD 9.4)
Gender, % male	60.9	50	42.1
Handeness, % right	87	92.9	89.5
Current employment status			
Working (paid), %	30.4	21.4	52.6
Retired, %	60.9	78.6	42.1
Other, %	8.6	–	5.3
Ethnicity			
White-british, %	100	100	89.5
Educational qualifications			
No qualifications, %	4.3	–	15.8
GSCSEs,BTEC intr.diplom. NVQs ls1-2, %	13.2	35.7	21.1
A-levels, International baccalaureate, NVQs lev 3, BTEC Nationals, %	30.4	14.3	21.1
Higher education, NVQs le 4–5, HNCs, BTEC professional diplomas, %	21.7	28.6	31.6
Masters, Doctoral degrees, BTEC AdvancedProfessional diplomas, %	30.4	21.4	10.5
*Cognition and IQ*			
MMSE- Total , mean (SD)	29.4 (3.4)	28.0 (1.7)	29.2 (0.8)
Estimated IQ on Test “g” C.Fair, mean (SD)	90.2 (14.1)	85.8 (17.0)	103.2 (12.0)
*Parkinson’s Disease characteristics*			
Disease duration, mean (SD),yr	9.9 (8.8)	7.7 (5.5)	N/A
Hoehn and Yahr stage, % 1/2/3/4/5	61.5 /26.9 /7.7 /0 /3.8	53.3 /20.0 /26.7 /0 /0	N/A
Levodopa therapy, % yes	80.8	86.7	N/A
*Psychopathology*			
BDI Total score (0–63), mean (SD)	8.1 (4.6)	13.0 (6.9)	3.7 (3.2)
PANSS Total Score (14–98), mean (SD)	14.1 (0.3)	16.4 (1.5)	14.0 (0.2)
CAARMS group			
Attenuated psychosis (subthresold), n (%)	–	13 (86.7)	–
Psychosis threshold, n (%)	–	2 (13.3)	–
CAARMS score equal or over 3, global rating scales			
UTC		–	
NBI , n (%)		4 (26.7)	
PA , n (%)		12 (80.1)	
DS , n (%)		2 (13.3)	
ADB		–	
SS		–	
GAF Scale-M (1–100), mean (SD)	92.1 (5.9)	81.4 (12.6)	98.0 (2.0)
GAF Scale-Disability (1–90), mean (SD)	82.0 (6.8)	73.6 (10.5)	89.5 (0.6)
GAF Scalw-Symptoms (1–90), mean (SD)	82.1 (7.9)	79.8 (11.4)	89.0 (0.6)
Apathy Evaluation Scale (18-items), mean SD	29.8 (6.7)	35.8 (7.5)	–
*Comorbidity mental illness*			
None, %	56.5	57.1	89.5
Depression, %	26.1	21.4	5.3
Anxiety, %	8.7	14.3	5.3
Missing, %	8.7	7.1	–
*Family history mental illness (depression)*			
None (%)	78.3	78.6	84.2
First relatives (%)	8.7	14.3	10.5
Other relatives (%)	8.7	–	5.3
Missing (%)	4.3	7.1	–

* Inclusion criteria: LIFETIME CAARMS scoring equal or over 3 in global and frequency scales.

Patients were recruited via the PD research clinic at the John van Geest Centre for Brain Repair (VGB). All patients met Queen Square Brain Bank Criteria for idiopathic PD (Gibb and Lees, 1988) and the patients remained on their usual medications during testing. Patients with dementia were excluded (mini-mental state score less than 24). In all cases, the patients anti-PD medication remained unchanged during testing, and was converted to a Levodopa Equivalent Dose (LED) using a standard approach (Tomlinson et al., 2010). The two patient groups did not significantly differ in the LED they received (p = .572). Before scanning, each of the participants underwent a general interview and clinical assessment using the Positive and Negative Symptom Scale (PANSS) (Kay et al., 1987), and the Global Assessment of Functioning (GAF) (Hall, 1995). The Apathy Evaluation Scale (AES) (Marin et al., 1991) was used to assess apathy. The Beck Depression Inventory (BDI) (Beck et al., 1996) was used to assess depressive symptoms during the last two weeks and IQ was estimated using the Culture Fair Intelligence Test (Cattell and Cattell, 1973) and cognitive function was measured using the mini-mental state examination (Folstein et al., 1975). Furthermore, all participants received a detailed clinical assessment from an experienced psychiatric nurse, including medical history and an in-house structured assessment of drug and alcohol intake, which could lead to further assessment for dependence if indicate.

All subjects had normal or corrected-to-normal visual acuity and were without any contraindications for MRI scanning. At the time of the study, none of the participants were taking antipsychotic medications or had drug or alcohol dependence. The study was approved by the Cambridgeshire 3 National Health Service research ethics committee. All subjects gave written informed consent in accordance with the Declaration of Helsinki.

### 2.2 Salience oddball task

**Fig. 1:**
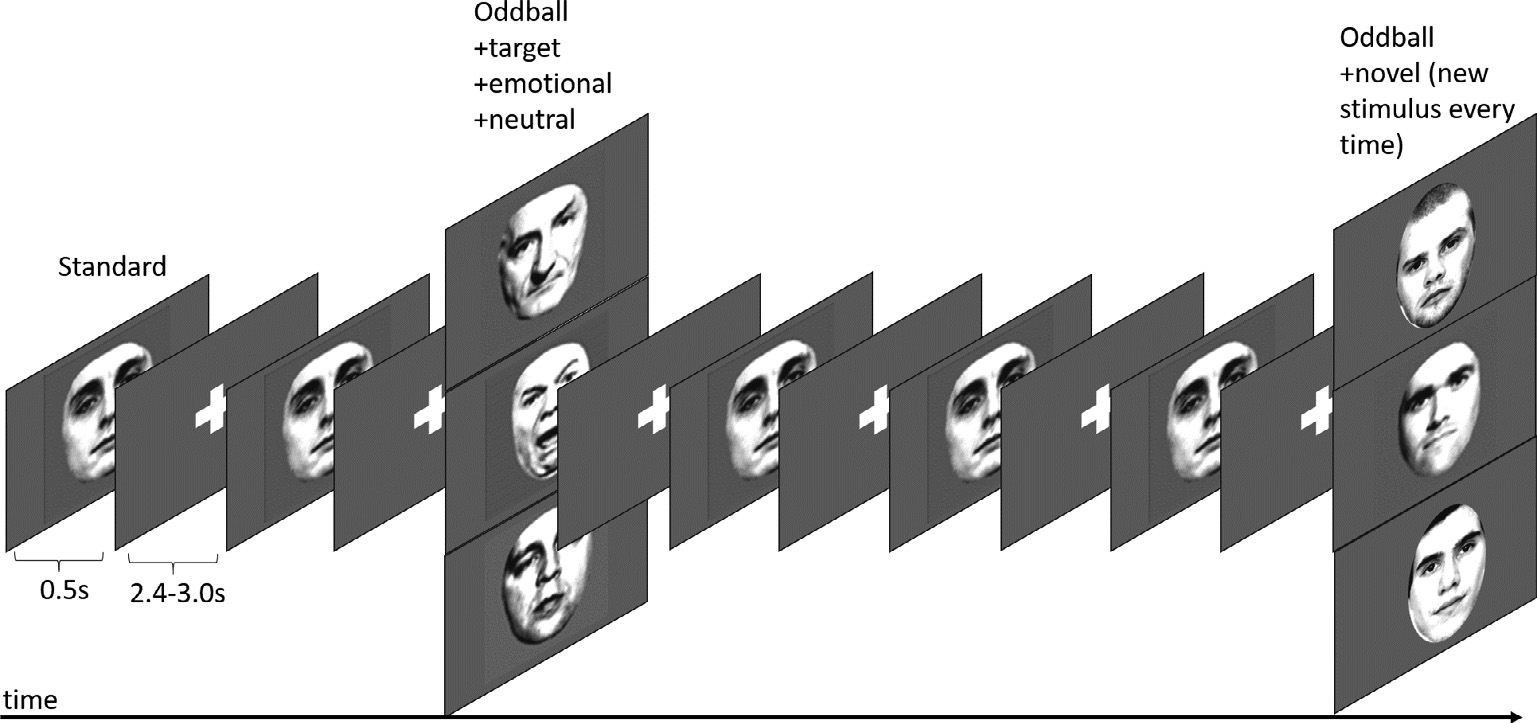
Task design (displayed for face stimuli only). During this visual oddball paradigm, participants are presented with a random series of greyscale images of faces and outdoor scenes. 66.6% of these were ‘standard’ images. The remaining 33.4% consisted of four types of rare or contextually deviant events, which were randomly intermixed with the standard stimuli; each occurred with a probability of 8.3%. These deviant events were: neutral stimuli that required a motor response (‘target oddball’); stimuli that evoked a negative emotional response (‘emotional oddball’, angry face or image of car crash); novel stimuli (‘novel oddball’, different neutral images that appear only once); and neutral stimuli (‘neutral oddball’, neutral image of face or scene). In the current study, we were only interested in the contrast between negative emotional and neutral oddball stimuli.

We used a visual oddball paradigm (Bunzeck et al., 2007) (Fig. 1) in order to investigate negative emotional salience (see (Bunzeck and Düzel, 2006, Knolle et al., 2018) for depiction of paradigm). A detailed description of the paradigm can be found in our previous work (Knolle et al., 2018): in summary, participants saw a series of greyscale images of faces and outdoor scenes. 66.6% of these were ‘standard’, neutral images. Then four types of deviating images were randomly intermixed with these; each type occurred with a probability of 8.3%. These deviant events were: stimuli that evoked a negative emotional response (‘emotional oddball’, angry face or image of car crash); neutral stimuli that required a motor response (‘target oddball’); neutral stimuli that presented a novel image every time they appeared (‘novel oddball’); and neutral stimuli of the same face or scene that did not require a motor response or contained negative/positive emotional valence (‘neutral oddball’). All participants completed 3 runs of 240 trials each (160 standard trials, and 20 oddball trials each of target, neutral, emotional and novel stimuli), resulting in a total of 720 trials. The task contained 50% faces and 50% outdoor scenes, to avoid category-specific habituation and to make stimulus exploration generalisable to different visual stimuli. The category switched once per run. The order in which the visual categories occurred was counterbalanced across participants. Participants were introduced to the target stimulus prior to the experimental session for 4.5 s, and they were required to make a simple button press in response using their left or right index finger to each of its subsequent appearances during the experiment within the fMRI-scanner. Participants used their preferred or less affected hand to press the buttons on the button box for the target picture. No motor responses were associated with any of the other stimulus types.

During the fMRI-experiment, the pictures were presented for 500 ms followed by a white fixation cross on a grey background (grey value = 127) using an inter-stimulus interval (ISI) of 2.7 s. ISI was jittered with ± 300 ms (uniformly distributed). All of the stimuli were taken from Bunzeck and Düzel (Bunzeck and Düzel, 2006). The scalp hair and ears of faces were removed artificially; the outdoor scenes did not include faces. All pictures were grey scaled and normalised to a mean grey value of 127 (SD 75). The pictures were projected on to the centre of a screen, and the participants watched them through a mirror mounted on the head coil, subtending a visual angle of about 8°.

In the current study, we focussed on negative emotional salience, this contrast was the most robust in terms of generating within and between group brain activations in our previous study in young adults with first-episode psychosis (Knolle et al., 2018). We contrasted activation associated with the emotional and neutral oddball stimuli. Both types of stimuli have the same frequency. This contrast, therefore, allowed us to examine brain responses to the negative emotional valence (‘emotional’ vs. ‘neutral’) of a salient event. In contrast, the classical oddball effect was sought by looking at the contrast between the neutral oddball and standard stimuli, which is based on frequency differences.

### 2.3 Behaviour analysis

An analysis of variance (ANOVA) was used to investigate group differences in responses to the target stimuli (i.e. button presses) as well as reaction times. Behavioural data were analysed using SPSS 21 (IBM Corp.).

### 2.4 Neuroimaging acquisition and analysis protocol

Data was collected using a Siemens Magnetom Trio Tim syngo MR B17 operating at 3 T.

We used a previously described protocol for the acquisition of the functional imaging data (Knolle et al., 2018). We acquired gradient-echo echo-planar T2*-weighted images depicting BOLD contrast from 27 non-contiguous oblique axial plane slices to minimise signal drop-out in the ventral regions. Images of the whole-brain were not always retrieved, depending on head size; the superior posterior part of the cortex was not always imaged (see Supplementary Fig. 1, for examples of registration). We used the following setup: relaxation time (TR): 1620 ms; echo time (TE): 30 ms; flip angle (FA): 65°; in-plane resolution: 3 × 3 mm; matrix size: 64 × 64; field of view (FoV): 192 × 192 mm; and bandwidth: 2442 Hz/px. We acquired a total of 437 volumes per participant (27 slices each of 3 mm thickness, inter-slice gap 1.5 mm). The first five volumes were discarded to allow for T1 equilibration effects.

We used FSL software (FMRIB’s Software Library, www.fmrib.ox.ac.uk/fsl) version five to analyse the functional data. Participants’ data (first-level analysis) were processed using the FMRI Expert Analysis Tool (FEAT). For each subject all three experimental runs were pre-processed separately using the following procedure: Functional images were realigned, motion corrected (MCFLIRT (Jenkinson et al., 2002)) and spatially smoothed with a 8 mm full-width half-maximum Gaussian kernel. A high-pass filter was applied (120 s cut-off). All images were registered to the whole-brain echo-planar image (EPI) (i.e., functional image with the whole-brain field of view; sequence parameters: number of volumes: 3; number of slices: 40 with a slice thickness of 3 mm and an interslice gap of 1.5 mm; order: interleaved, descending; TR: 2380 ms, TE: 30 ms, FA: 65°, matrix size: 64 × 64; FoV: 192 × 192 mm; in-plane resolution: 3x3 mm), and then to the structural image of the corresponding participant (MPRAGE; sequence parameters: TR: 2300 ms, TE: 2.98 ms, flip angle: 9°, spatial resolution: 1 × 1 × 1 mm) and normalised to an MNI template, using linear registration with FSL FLIRT.

The five explanatory variables (EVs) that we used were the onset times of the standard, target, emotional, novel and neutral pictures. They were modelled as 1 s events and convolved with a canonical double-gamma response function. We added a temporal derivative to the model to take into account possible variations in the haemodynamic response function. To capture residual movement-related artefacts, six covariates were used as regressors of no interest (three rigid-body translations and three rotations resulting from realignment). We used four contrasts: target-neutral, emotion-neutral, novel-neutral, and neutral-standard, although the last contrast represents a frequency contrast on neutral images. However, as pointed out before, in this study we focussed on the contrast of emotional-neutral, and report results on the other contrasts for completeness only. In the “second-level” within-subject analysis, we combined the data for the three experimental runs for each participant using FEAT with Fixed Effects. This step was specifically done to average the three experimental runs for each participants. In the third-level (group) analysis, we conducted an ANOVA using FEAT, mixed effects (FLAME (FMRIB's Local Analysis of Mixed Effects) modelling and estimation, a two-stage process using Bayesian modelling and estimation), on our contrast of interest (negative emotional oddball vs. neutral oddball)). We used cluster-based statistical approaches (TFCE) with family wise error corrections.

### 2.5 Region of interest analysis for all voxels within one cluster

**Fig. 2:**
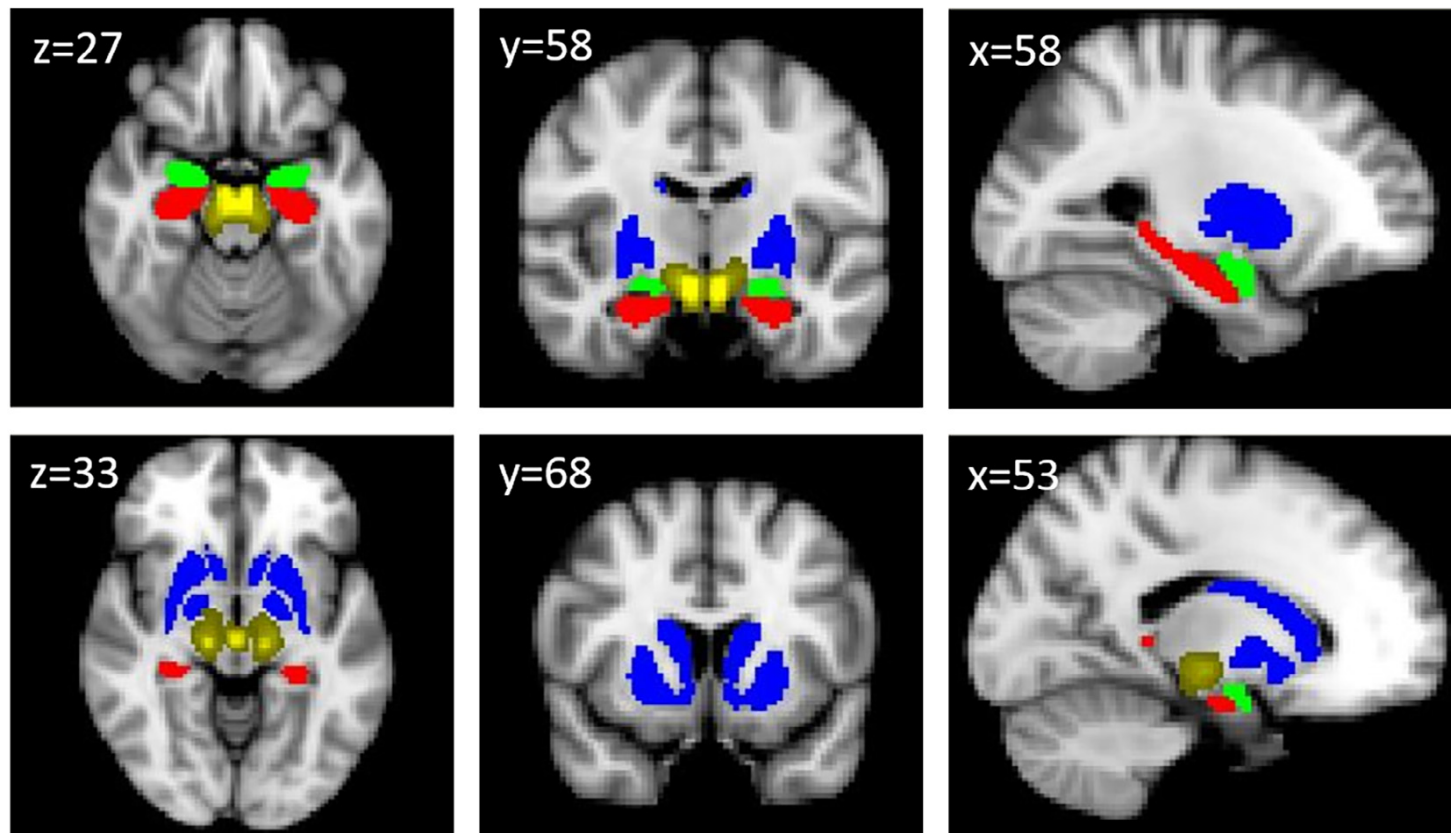
Display of masks used for the region of interest analysis: the bilateral striatum (blue, 3039 voxels), the hippocampus (red, 1033 voxels), the amygdala (green, 505 voxels), and the substantia nigra/VTA (yellow, 645 voxels). (For interpretation of the references to colour in this figure legend, the reader is referred to the web version of this article.)

For our main analysis, we pursued a ROI approach: For our salience type of interest – negative emotional salience – our primary hypothesis involved four regions of interest that have been found to be most active in this paradigm (Knolle et al., 2018). These four regions included the dopaminergic midbrain (substantia nigra/ventral tegmental area (VTA)), the ventral and dorsal striatum, the hippocampus and amygdala bilaterally. The mask for the dopaminergic midbrain region was generated using the probabilistic atlas of Murty and colleagues (Murty et al., 2014) and has been used successfully in our own previous work (Ermakova et al., 2018, Knolle et al., 2018). Masks for all other regions were anatomically derived using the Harvard-Oxford subcortical structural atlas supplied with FSL. We defined the dorsal and ventral striatum as a combination of what is in the Harvard-Oxford subcortical atlas labelled as caudate, putamen and nucleus accumbens. The individual regions contained voxel sizes as follows: bilateral striatum (3039 voxels), hippocampus (1033 voxels), amygdala (505 voxels), and substantia nigra/VTA (645 voxels). See Fig. 2 for display of ROI. For planned group comparisons, we extracted contrast values (contrast of parameter estimates, or COPEs in FSL) for each individual from all the voxels within each of the four ROIs. We furthermore used the Featquery application in FSL to extract parameter estimates for individual event types within regions of interest for analysis presented in the supplementary materials. Average COPE values per region of interest were entered into a multivariate analysis of variance to compare groups.

For completeness and to match the analyses performed in our previous work using the same paradigm (Knolle et al., 2018) we conducted additional permutation analyses on the ROI and whole brain. Analysis steps and results are presented in the supplementary materials.

### 2.6 Resting cerebral blood flow

Interpretation of BOLD activation effects is complicated by difficulties in assessing whether any results are truly due to differences in evoked activation, or to baseline cerebral blood flow (CBF) (perfusion) differences “at rest” (Fleisher et al., 2009, Simon and Buxton, 2015). CBF could be altered by disease course or medication, as dopaminergic drugs act directly on the blood vessels and lead to vasodilation which increases CBF (Leenders et al., 1985). We therefore assessed resting CBF at baseline for all participants except for one PD patient without psychotic symptoms. For this assessment, we used a continuous arterial spin labelling (cASL) protocol described in Wang and colleagues (Wang et al., 2005) and adopted in other studies (Viviani et al., 2009). We used the following setup: relaxation time: 4000 ms; echo time: 17 ms; sequence: gradient-echo echo-planar imaging sequence with anterior-to-posterior phase encoding; multi-slice mode: interleaved; number of images: 120 with and without labelling; flip angle: 90°; in-plane resolution: 3.8 × 3.8 × 6 mm; slice thickness: 6 mm; matrix size: 64 × 64; field of view: 249 × 249 mm; and bandwidth: 2442 Hz/px. We inserted a 1 s delay between labelling pulse and image acquisition. We used the SPM2 package (Wellcome Department of Cognitive Neurology, London; online at http://www.fil.ion.ucl.ac.uk) for realignment and stereotactic normalization to an EPI template (Montreal Neurological Institute, resampling size: 2 × 2 × 2 mm). Using the Perf_resconstruct_V02 SPM add-on software by Rao and Wang (Department of Radiology and Center for Functional Neuroimaging at University of Pennsylvania; online at http://www.cfn.upenn.edu/perfusion/software.htm), we reconstructed resting CBF values. We then used a ‘simple subtraction’ method (Wang et al., 2003). All volumes were smoothed using an isotropic Gaussian kernel of full width half-maximum (FWHM) of 8 mm prior to the analysis. We used the SPM PET basic models setup to generate our group statistics and then a one-way ANOVA with an explicit mask and an ANOVA normalisation. The significance threshold was set at *p=*.05, which was corrected for multiple comparisons by using the false discovery rate. We also used the Marsbar toolbox to extract mean CBF for our regions of interest and then employed those values as covariates in our planned group comparisons for the task activations. Statistical analyses were generated using SPSS 21 (IBM Corp.).

### 2.7 Exploratory correlation analysis of symptom scores, brain responses and medication

We conducted exploratory two-way Pearson correlations per group between medication (LED), symptom scores (BDI, GAF, AES, CAARMS total) and brain activations (resting CBF and BOLD responses to four regions of interest).

### 2.8 Movement differences during fMRI scan

The task was split into 3 runs of 11.5 min. Runs in which movement exceeded 3 mm on average or 10 mm maximum were excluded from the analysis. We only included participants with at least two runs. We identified two PD patients without psychotic symptoms that had movement exclusion criterion in two out of three runs and so they were excluded from all the analyses.

We compared the maximum and mean movement across the three runs in two separate repeated measure ANOVAs (Table 2). We did not find any significant group, run or interactions effect, neither for mean movement nor for maximum movement (all p > 0.1).

**Table 2:** Mean and maximum movement across testing blocks and groups (in mm).

	Group	Mean (SD)	Max (SD)
Run 1	PD-Psychosis	0.79 (0.63)	2.22 (1.58)
	PD + Psychosis	0.74 (0.40)	2.08 (1.31)
	Controls	0.46 (0.24)	1.27 (0.82)
Run 2	PD-Psychosis	0.73 (0.63)	2.21 (1.87)
	PD + Psychosis	0.80 (0.54)	1.98 (1.24)
	Controls	0.54 (0.43)	1.60 (1.45)
Run 3	PD-Psychosis	0.86 (0.64)	2.70 (2.02)
	PD + Psychosis	0.68 (0.51)	2.01 (1.48)
	Controls	0.68 (0.83)	1.68 (1.56)

PD-Psychosis: PD patients without psychosis, PD + Psychosis: PD patients with psychosis.

## 3 Results

### 3.1 Behavioural results

Throughout the task, participants were asked to press a button in response to two target pictures - one for the face stimuli and one for the scene stimuli. This ensured that participants maintained their attention throughout the task. In two separate repeated measure ANOVAs, we analysed the number of button presses and the reaction times in response to both target pictures together (Supplementary Fig. 2A/B and 3, respectively). We found a significant effect for the number of misses across runs (F(2) = 3.82p = .025, 68.3%power), but no group effect or interaction. Bonferroni corrected post hoc-tests revealed that participants missed marginally more button presses in the third compared to the second run (p = .059). On average, participants failed to press the button on 6 target trials per run (mean run 1 = 5.78 (SD = 2.4); mean run 2 = 5.45 (SD = 2.4); mean run 3 = 6.4 (SD = 3.1)). Furthermore, we found a significant effect for reaction time across runs (F(2) = 6.31p = .003, 88.9%power), but no group effect or interaction. Bonferroni corrected post hoc-tests revealed that participants reacted significantly faster to target images in run 1 compared to run 2 (p = .014) and run 3 (p = .019). On average, participants required between 500 and 600 ms (mean run1 = 0.531 (SD = 0.1); mean run2 = 0.555 (SD = 0.1); mean run3 = 0.560 (SD = 0.1)) to make a response, which is consistent with previous our findings (Knolle et al., 2018).

### 3.2 Imaging results

#### 3.2.1 Group analysis of resting cerebral blood flow

The one-way ANOVA on resting CBF data did not reveal any significant group differences.

#### 3.2.2 fMRI activation to emotional salience

**Fig. 3:**
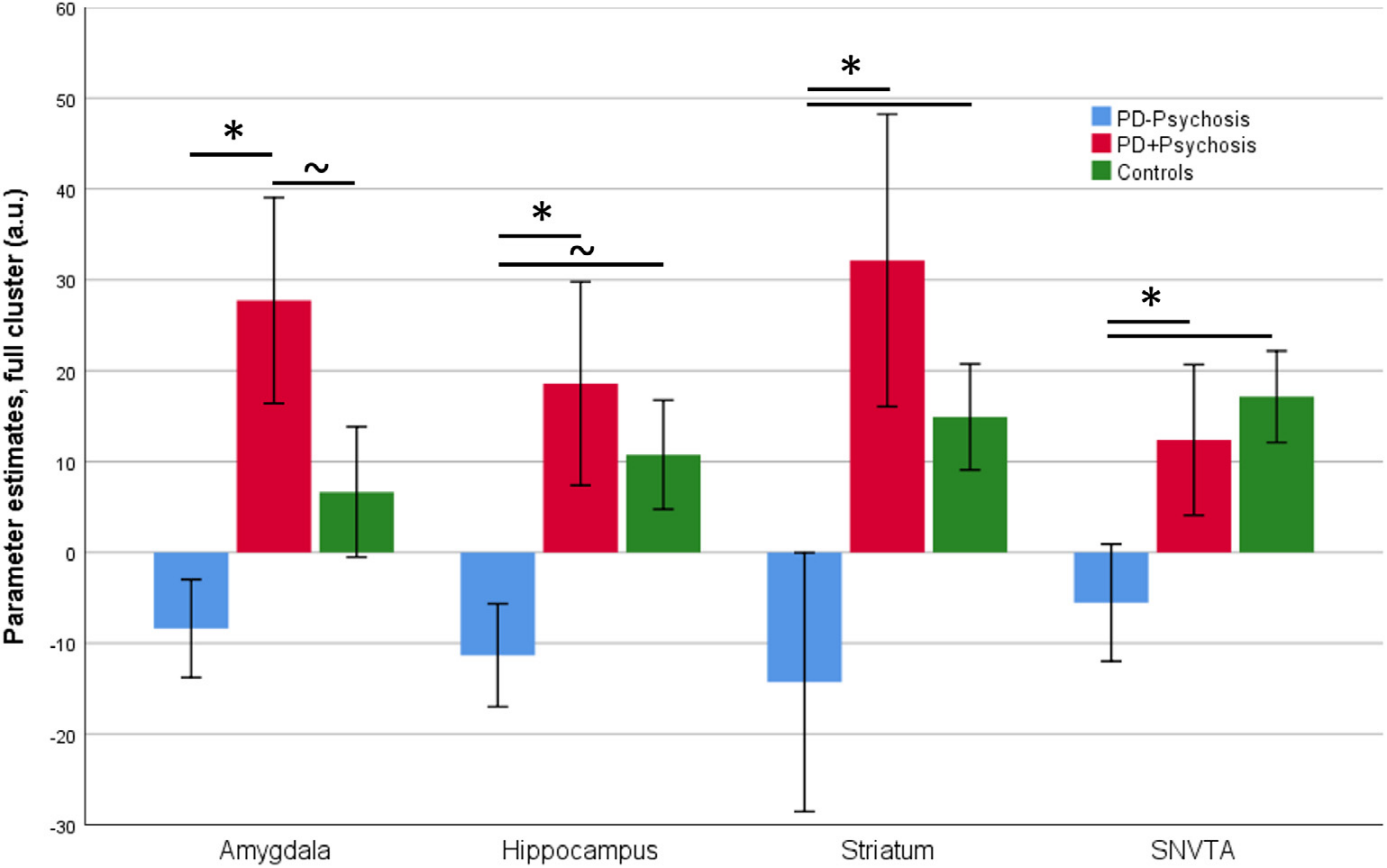
Bar chart shows mean contrast (COPEs, FSL) values extracted from all voxels of each region of interest and significant group effects (values uncorrected for covariates). Error bars show ± 1 SE. *p < .05. PD-Psychosis: PD patients without psychosis, PD + Psychosis: PD patients with psychosis.

In our main analysis, we investigated potential group differences in emotional salience related activation, while controlling for resting CBF. We extracted the mean BOLD activation (COPE, contrast of parameter estimates between neutral and emotional oddballs) as well as the mean resting CBF from each individual region used in the ROI cluster. We conducted a multivariate analysis of variance to determine whether there were any statistically significant differences between the Parkinson’s patients with psychotic symptoms, Parkinson’s patients without psychotic symptoms and healthy controls on the BOLD activation per region controlling for CBF in the corresponding region (Fig. 3).

The multivariate test revealed a significant group effect on brain activation in response to negative emotional salience within the ROIs, controlling for resting CBF in ROIs respectively, Pillai’s V = 0.32, F(8,88) = 2.08p = .046. Tests of between-subject effects furthermore revealed significant group effects in the amygdala signal bilaterally, F(2,46) = 5.83p = .006, partial η2 = 0.20, 85.0% power, the hippocampus bilaterally, F(2,46) = 3.31p = .016, partial η2 = 0.16, 74.2% power, the striatum, F(2,46) = 5.17p = .009, partial η2 = 0.18, 80.2% power, and the substantia nigra/VTA, F(2,46) = 3.52p = .009, partial η2 = 0.13, 62.8% power. As a control analysis, we ran the multivariate analysis without controlling for CBF. These results (presented in the supplementary materials) are very similar and supports the conclusion that the effects seen were are not driven by differences in the CBF.

In the amygdala, we found bilaterally significantly greater (p = .001) activation in PD patients with psychotic symptoms (mean^1^: 29.38, SE 9.2) compared to those without psychotic symptoms (mean^1^: −10.52, SE 7.3). Controls (mean^1^: 6.61, SE 8.6) differed marginally from PD patients with psychotic symptoms (p = .094), but not from those without psychotic symptoms.

We found significantly greater activation in the hippocampus bilaterally (p = .007) in PD patients with psychotic symptoms (mean^1^: 19.41, SE 9.0) compared to those without (mean: −12.60, SE 7.1). Controls (mean^1^: 10.39, SE 8.5) had marginally significantly greater activity compared to PD patients without psychotic symptoms (p = .052) but they did not significantly differ from PD patients with psychotic symptoms.

In the striatum we found significantly greater (p = .003) activation in PD patients with psychotic symptoms (mean^1^: 35.71, SE 14.0) compared to those without (mean^1^: −20.39, SE 11.0). Controls (mean^1^: 16.79, SE 13.3) differed significantly from those patients without psychotic symptoms (p = .044) but not from those with psychotic symptoms.

In the substantia nigra/VTA we found significantly greater (p = .026) activation in PD patients with psychotic symptoms (mean^1^: 16.66, SE 8.0) compared to those without (mean^1^: −6.63, SE 6.3). Controls (mean^1^: 14.34, SE 7.5) differed significantly from those patients without psychotic symptoms (p = .045) but not from those with psychotic symptoms.

### 3.3 fMRI activation to novelty salience

For completeness, we conducted the same analysis as reported for emotional salience in novelty, as it is of theoretical interest in models of psychosis. We did not find any significant results, neither within nor between groups. We did not analyse for targetness as the participants did not respond to the target image in roughly 20% of the events and similarly for rareness as this is a simple frequency response without specific salient content.

### 3.4 Results for exploratory correlations of symptom scores, brain responses and medication

**Fig. 4:**
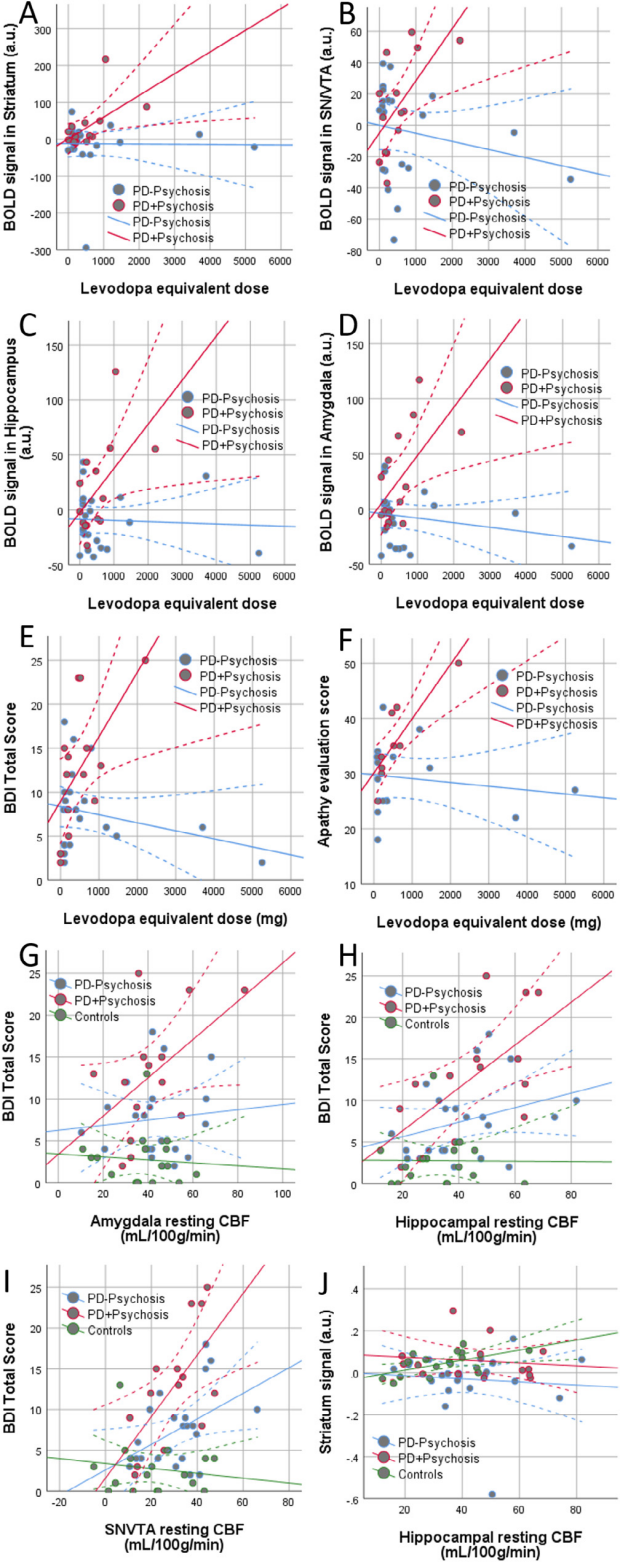
Scatter plots show correlations that yielded a significant result in at least one group. In PD patients with psychotic symptoms, we found a positive correlation between LED and the BOLD activation in the ROIs (A: bilateral amygdala: r = 0.603, p = .023, B: bilateral hippocampus: r = 0.560, p = .037, C: substantia nigra/VTA: r = 0.631, p = .016, D: striatum: r = 0.573, p = .032). LED was positively correlated to BDI score (E: r = 0.591, p = .025) and apathy score (F: AES, r = 0.849, p = .004); and BDI score was positively correlated to resting CBF bilaterally in the amygdala (G: marginally significant: r = 0.515, p = .059), the hippocampus (H: r = 0.631, p = .015) as well as the substantia nigra (I: r = 0.646, p = .013). In patients without psychotic symptoms, we found that the BDI score was positively correlated to resting CBF in the substantia nigra (I: r = 0.450, p = .036). Only in controls we found a significant correlations between BOLD activation and resting CBF; BOLD signal in the striatum was significantly correlated to bilateral resting CBF in the hippocampus (J: r = 0.567, p = .018).

All significant results from the Pearson’s correlations are presented in Fig. 4. Importantly, in PD patients with psychotic symptoms, we found a positive correlation between LED and the BOLD activation in the ROIs (bilateral amygdala: r = 0.603, p = .023, bilateral hippocampus: r = 0.560, p = .037, substantia nigra/VTA: r = 0.631, p = .016, striatum (marginally significant): r = 0.514, p = .060). We did not find the same correlation in patients without psychotic symptoms. Furthermore, in patients with psychotic symptoms, LED was positively correlated to BDI score (r = 0.591, p = .025) and apathy score (AES, r = 0.849, p = .004). In the same patients, we found, however, that the BDI score was positively correlated to resting CBF bilaterally in the hippocampus (r = 0.631, p = .015), the amygdala (marginally significant: r = 0.515, p = .059) as well as the substantia nigra (r = 0.646, p = .013). Furthermore, we did not find any significant correlations between symptom scores (AES, GAF disability and BDI scores) and BOLD responses to negative emotional stimuli, except for one significant positive correlation in the patients with psychotic symptoms, where higher apathy scores were related to greater BOLD scores in the striatum (r = 0.695, p = .038). We did not find any significant correlations between LED and resting CBF.

Pearson correlation analysis did not reveal any significant correlations between BOLD activation and resting CBF in the patient groups. In the controls however, we found significant correlation between the BOLD signal in the striatum and resting CBF in the striatum, (r = 0.554, p = .021), hippocampus (r = 0.526, p = .030) and substantia nigra/VTA (marginal effect r = 0.458, p = .060).

In order to compare the correlations within each group against each other, we used the Fisher r-to-z transformation. This allows us to test whether the correlations in PD patients with psychotic symptoms were significantly different from the correlations in the other groups (see Supplementary Table 1). We found that the correlations of LED and BOLD activation as well as symptom scores were significantly different between the two patient groups. Correlations between BDI and resting CBF in patients with psychotic symptoms differed significantly from those in controls, but not from the other patient group.

## 4 Discussion

In the current study we investigated negative emotional salience in PD patients with and without psychotic symptoms and compared them to healthy controls. Based on previous studies and the literature (Ermakova et al., 2018, Garofalo et al., 2017, Knolle et al., 2018), we hypothesised there would be altered brain activity in the striatum, dopaminergic midbrain (i.e., substantia nigra/VTA), hippocampus and amygdala in both patients’ groups compared to control subjects, with an intermediate alteration in PD patients without psychotic symptoms. The study was not designed to differentiate between emotional scene and emotional face salience, it is possible that the group differences were mainly driven by one of these categories. However, as we were interested in emotional salience processing in general we analyse the two categories jointly, and ignored their individual contribution to the effect.

In line with our hypothesis, we found significant differences between the patient groups. PD patients with psychotic symptoms had strongly enhanced brain responses in all four regions of interest (i.e., the striatum, the substantia nigra/VTA, the hippocampus and the amygdala) compared to PD patients without psychotic symptoms. PD patients with psychotic symptoms showed slightly stronger, but insignificantly different BOLD signals compared to controls in all regions bar the substantia nigra/VTA, suggesting maintained emotional salience processing in the patients with psychotic symptoms. PD patients without psychotic symptoms, however, differed significantly (or at least marginally) from controls in all four regions, showing a generally altered ability to process emotionally salient stimuli. The finding of abnormal salience associated brain activation in PD patients without psychotic symptoms matches the findings in the literature regarding the deficiencies in emotional processing in PD patients (Moonen et al., 2017, Péron et al., 2012). Salience associated brain activation in PD psychosis may appear to be normal when compared to controls, but when compared to PD patients without psychosis there is a clear difference, PD psychosis appears to be associated with a relative increase in salience-associated brain activation. Here we provide a speculative explanation for these complex but very interesting results.

The finding that PD patients without psychosis show an altered ability to process emotionally salient stimuli is in concert with the literature reporting deficiencies in emotional processing in PD patients (without psychotic symptoms) using ERP and behavioural tasks (for review see (Moonen et al., 2017, Péron et al., 2012)). The PD patients with psychotic symptoms show, despite the general deficits in emotional processing inherent to the disorder, a pattern of activation which is comparable to that of controls. These results might suggest that PD patients with psychosis show aberrantly enhanced or altered salience processing which overrides the emotional processing deficits and leads to an apparent compensation of the emotional processing deficits. This interpretation, however, requires further experimental exploration.

Our study is the first to investigate emotional salience processing in PD patients with and without psychotic symptoms and controls. Importantly, our study controls for putative dopaminergic medication effects on the baseline BOLD signal strength by assessing the resting cerebral blood flow (resting CBF) in all groups. The current study reveals that PD patients with psychotic symptoms show a strongly enhanced response to salience in the striatum, substantia nigra/VTA, amygdala and hippocampus compared to PD patients without psychotic symptoms. The pattern of activation in the PD patients with psychotic symptoms is opposite to that which has been reported in patients with primary psychosis (Knolle et al, 2018). Interestingly, however, in this work we also found that the stronger the psychotic symptoms, the stronger the activation in response to emotional salience which is in line with the results in the PD patients with psychotic symptoms. Correspondingly, an exploratory analysis revealed that in PD patients with psychotic symptoms, the dose of dopaminergic medication as measured by LED, positively correlated with the BOLD activation in all ROIs (i.e., bilateral amygdala, bilateral hippocampus, substantia nigra/VTA). In addition, the dopaminergic medication dose was positively linked to measured depression (BDI) and apathy (AES), as well as negatively linked to global functioning (GAF disability). In patients without psychotic symptoms, we did not find any significant correlations between brain activation and psychopathology or medication, with controls also not showing any correlations between brain activation and psychopathology. There was importantly no significant difference in the overall medication dose between both patient groups. Our findings do though support early studies showing that dopaminergic medication is not the only, or even main, cause of psychotic symptoms in PD, but might function as an enhancer (for review see (Chang and Fox, 2016, Gallagher and Schrag, 2012)). We therefore suggest a potential imbalance in the interaction between medication dependent tonic dopamine levels and phasic dopamine responses to sensory inputs in PD patients with psychotic symptoms.

Our study indicates a link between the use of dopaminergic medication, processing alterations of salient stimuli as well as symptom scores. This is consistent with research showing that the administration of a dopamine agonist (pramipexole or ropinirole) in young medication-naïve PD patients led to an increase in aberrant motivational salience by facilitating arbitrary and illusory associations between stimuli and rewards, along with faster reaction times to task-irrelevant stimuli as well as a slight increase in psychotic like symptoms (Nagy et al., 2012). Further supporting evidence comes from a study by Poletti and colleagues (Poletti et al., 2012) which showed that in PD patients delusional jealousy was correlated with use of dopaminergic agonists, but not with any other medication or dementia. They also reported that delusional jealousy was independent of visual hallucinations, also assessed in the study, which were correlated with disease duration and levodopa therapy. The same group showed in a different study (Poletti et al., 2014) that aberrant salience, as assessed with the Aberrant Salience Inventory, correlated with dopaminergic treatment, especially levodopa. This suggests that the dopaminergic restoration of the early affected dorsal frontostriatal loop might lead to an overdose of the ventral loop which is relevant for salience processing (Cools et al., 2001, Poletti et al., 2014). This finding relates to the positive correlation we now report between the daily dose of medication (LED) and the activation in our ROIs.

In our study, we also found that only in PD patients with psychotic symptoms did the resting CBF within the four ROIs positively correlate with depression severity, linking higher depression severity with higher resting CBF. When comparing the correlations across groups, PD patients with psychotic symptoms were only significantly different from the correlations in healthy controls. As the correlations were stronger in patients with, compared to those without psychotic symptoms, the results provide some additional indication for the mechanistic link between risk factors like depression and PD psychosis. BOLD signal strength has been reported to depend on CBF levels (Simon and Buxton, 2015) but importantly we did not find that there were any significant differences in these parameters between groups. Therefore, it is unlikely that this correlation could fully explain the opposing signal between the two patient groups.

Our results are consistent with prior evidence that the use of dopaminergic medication is linked to the development of psychotic symptoms in PD patients (Zahodne and Fernandez, 2008). However, we still lack a full mechanistic explanation of why the use of dopaminergic drugs lead to psychotic symptoms in some patients but not in others. The aberrant salience hypothesis of psychosis suggests, first, that a dysregulated dopaminergic system in the mesolimbic system leads to the attribution of salience to otherwise irrelevant signals (Kapur, 2003); and, second, that these irrelevant signals are taken as valid information, and integrated by seemingly plausible top-down explanations, which supports the development of delusions and hallucinations.

With regard to the first prerequisite, PD patients show a clear dopaminergic pathology, which may involve dysregulation in some patients. Deficits in critical reasoning and accepting hasty cognitive explanations have often been reported in psychosis, mainly in schizophrenia but also other psychotic disorders (Garety et al., 2005, Lincoln et al., 2010). ‘Jumping to conclusions’ reflects a bias in critical reasoning where individuals draw a conclusion based on too little information for making an informed decision. In psychosis, ‘jumping to conclusions’ is considered a trait contributing to developing delusions (Garety and Freeman, 2013), as individuals who jump to conclusions might be prone to accepting implausible ideas and disregard alternative explanations. Djamshidan and colleagues were able to detect a bias in generating and accepting abnormal explanations for aberrantly salient stimuli in medicated (Djamshidian et al., 2012) and unmedicated (de Rezende Costa et al., 2016) PD patients. We speculate that this could relate to a cortical pathology, which is now well recognised to be a common feature in Parkinson’s disease (Kövari et al., 2003, Mattila et al., 2000). In the supplement of our current study, we also report some exploratory ‘jumping to conclusions’ results collected in a reduced sample from our cohort of patients and controls. The current state of the data collection does not allow a reliable interpretation, however, it provides a preliminary indication that PD patients with psychotic symptoms do show some impairments in this task and present a jumping to conclusions bias, which was not present in PD patients without psychotic symptoms or controls. We therefore suggest that the development of psychotic symptoms in PD patients may result from a combination of aberrantly enhanced salience signals in the striatal-hippocampal-midbrain circuits and deficient cognitive reasoning (possibly cortical) processes. A similar view is presented in Poletti and Bonuccelli (2013) who argue that impaired salience processing and a deficit in higher-order control mechanisms (such as the ‘jumping to conclusions’ bias) potentially giving rise to psychotic symptoms.

In conclusion, our study provides evidence for the first time that aberrant striatal, hippocampal and amygdala signalling during processing of non-motivational salient stimuli differentiates PD patients with and without psychotic symptoms. The results suggest that enhanced signalling in these regions, possibly leads to the development of psychotic symptoms, in a similar way as that proposed in the ‘aberrant salience hypothesis’ of psychosis.

## CRediT authorship contribution statement

**F. Knolle:** Visualization, Formal analysis, Methodology, Investigation, Funding acquisition, Writing - review & editing, Writing - original draft. **S. Garofalo:** Writing - review & editing. **R. Viviani:** Methodology, Writing - review & editing. **A. Justicia:** Data collection. **A.O. Ermakova:** Data collection, Methodology. **H. Blank:** Methodology. **G.B. Williams:** Methodology. **G. Arrondo:** Writing - review & editing. **P. Ramachandra:** Writing - review & editing. **C. Tudor-Sfetea:** Writing - review & editing. **N. Bunzeck:** Writing - review & editing. **E. Duezel:** Writing - review & editing. **T.W. Robbins:** Conceptulization, Writing - review & editing. **R.A. Barker:** Conceptulization, Writing - review & editing. **G.K. Murray:** Conceptulization, Project administration, Supervision, Methodolgy, Writing - review & editing, Funding acquisition.

## Footnotes

1 All mean values adjusted for CBF.
