# Supplementary material for: Altered subcortical emotional salience processing differentiates Parkinson’s patients with and without psychotic symptoms
Source: Neuroimage Clin. 2020 May 30;27:102277. doi: 10.1016/j.nicl.2020.102277 (PMC7298672; doi:10.1016/j.nicl.2020.102277)
Supplement: Supplementary data 1 [file mmc1.docx]

---------------------------------------------Supplementary materials--------------------------------------------

**Altered subcortical emotional salience processing differentiates Parkinson’s patients with and without psychotic symptoms**

Knolle F., [Garofalo](https://www.ncbi.nlm.nih.gov/pubmed/?term=Garofalo%20S%5BAuthor%5D&cauthor=true&cauthor_uid=28484422), S., Viviani R., [Justicia](https://www.ncbi.nlm.nih.gov/pubmed/?term=Justicia%20A%5BAuthor%5D&cauthor=true&cauthor_uid=28484422), A, [Ermakova](https://www.ncbi.nlm.nih.gov/pubmed/?term=Ermakova%20AO%5BAuthor%5D&cauthor=true&cauthor_uid=28484422), A.O., Blank H., Arrondo, G.,  [Ramachandra](https://www.ncbi.nlm.nih.gov/pubmed/?term=Ramachandra%20P%5BAuthor%5D&cauthor=true&cauthor_uid=28484422), P., ^,^[Tudor-Sfetea](https://www.ncbi.nlm.nih.gov/pubmed/?term=Tudor-Sfetea%20C%5BAuthor%5D&cauthor=true&cauthor_uid=28484422), C., Bunzeck, N., Duezel, E., [Robbins](https://www.ncbi.nlm.nih.gov/pubmed/?term=Robbins%20TW%5BAuthor%5D&cauthor=true&cauthor_uid=28484422),T.W.,  [Barker](https://www.ncbi.nlm.nih.gov/pubmed/?term=Barker%20RA%5BAuthor%5D&cauthor=true&cauthor_uid=28484422), R.A, and [Murray](https://www.ncbi.nlm.nih.gov/pubmed/?term=Murray%20GK%5BAuthor%5D&cauthor=true&cauthor_uid=28484422), G.K.


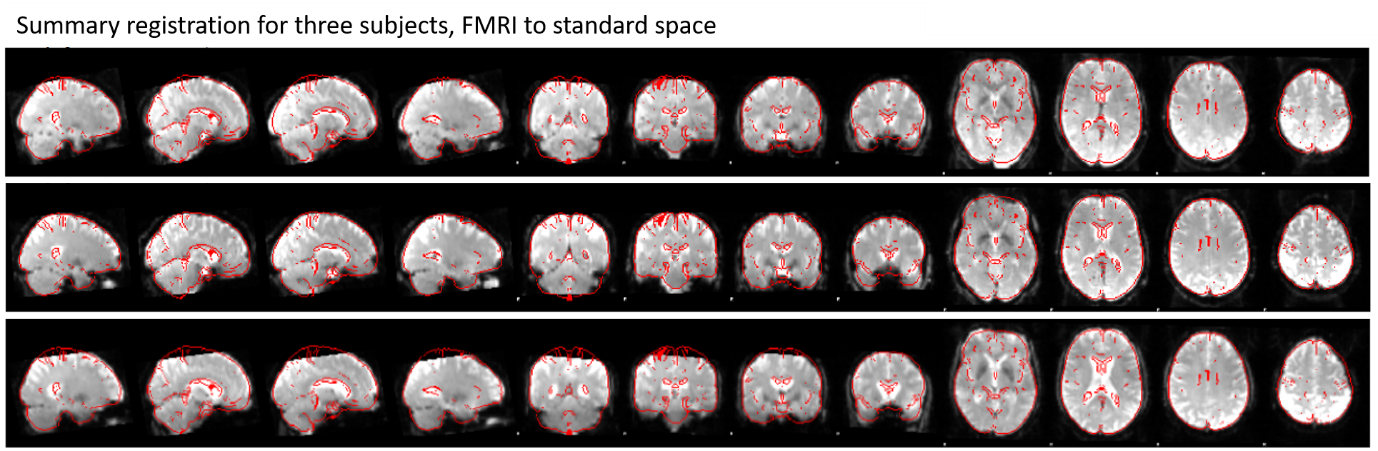


**Supplementary Figure 1**. Summary registration for three subjects. The top row presents a subject with average head size, the middle row a subject with a small head size and the last row a subject with large head size.

***Supplementary Table 1: Statistical comparison between the correlations***

|  | **Correlation between** | **PD without psychotic symptoms** | **Healthy controls** |
| --- | --- | --- | --- |
| **PD without psychotic symptoms** | LED and BOLD activation in   - Bilateral amygdala - Bilateral hippocampus - Substania nigra/VTA - striatum | ***Z, p***  2.62, 0.009  2.29, 0.022  2.13, 0.03  2.15, 0.03 | N.A. |
|  | LED and psychiatric symptom scores   - BDI - AES - GAF | 2.41, 0.02  2.43, 0.02  -1.4, 0.08* | N.A. |
|  | BDI and resting CBF in   - Bilateral amygdala - Bilateral hippocampus - Substania nigra/VTA | 1.26, 0.22  1.11, 0.26  0.75, 0.45 | 1.6, 0.055*  1.87, 0.03*  2.28, 0.02 |

*one-tailed

***Voxelwise permutation analysis – Region of interest and whole brain***

For permutation analysis at the level of voxel-based resolution, we used a mask that combined the bilateral striatum, hippocampus and amygdala, and the substantia nigra/VTA within one ROI, with a total of 5222 voxels. For estimation of group comparison (higher level, or “third-level”) statistics, we used permutation testing utilising the FSL randomise tool within our ROIs mask and the whole brain (the whole brain analysis was a complementary analysis done for completeness), with threshold-free-cluster enhancement, which enhances cluster-like structures but remains fundamentally a voxel-wise statistical testing method (Winkler et al., 2014). We used 5000 permutations and report significant results at p=0.05 or less following family-wise error correction for multiple comparisons, using the variance smoothing option (3mm) as recommended for experiments with small to modest sample sizes, as is common in fMRI research (Nichols and Holmes, 2002). We applied this method to our combined ROI and the whole brain. We used fslmeants to extract means from voxels revealing a significant group difference; we then used the extracted values purely for visualization of the group effect.

*Voxelwise permutation analysis on negative emotional salience BOLD activation*

In our ROI analysis, we found two clusters within the striatum, the left putamen and left pallidum, in which groups significantly differed (Supplementary Table 2, Supplementary Figure 2 A).

Furthermore, a complementary whole brain analysis, which was done for completeness, revealed significant differences in the parietal operculum, the supramarginal gyrus, planum temporale and pre- and postcentral gyrus (Supplementary Table 2, Figure 3 A).

For visualisation purposes only, we extracted parameter estimates from the significant clusters, which allows us to observe the directionality of the effect and the group behaviour (Supplementary Figure 2 B and C).

Additionally, we extracted the parameter estimates separately in response to emotional and neutral oddballs, as the conditions which define the contrast of interest. These are presented in the supplementary materials (Supplementary Figure 2) for all regions within the ROI. The parameter estimates indicate the potential drivers of the COPE (contrast of parameter estimates) effect.


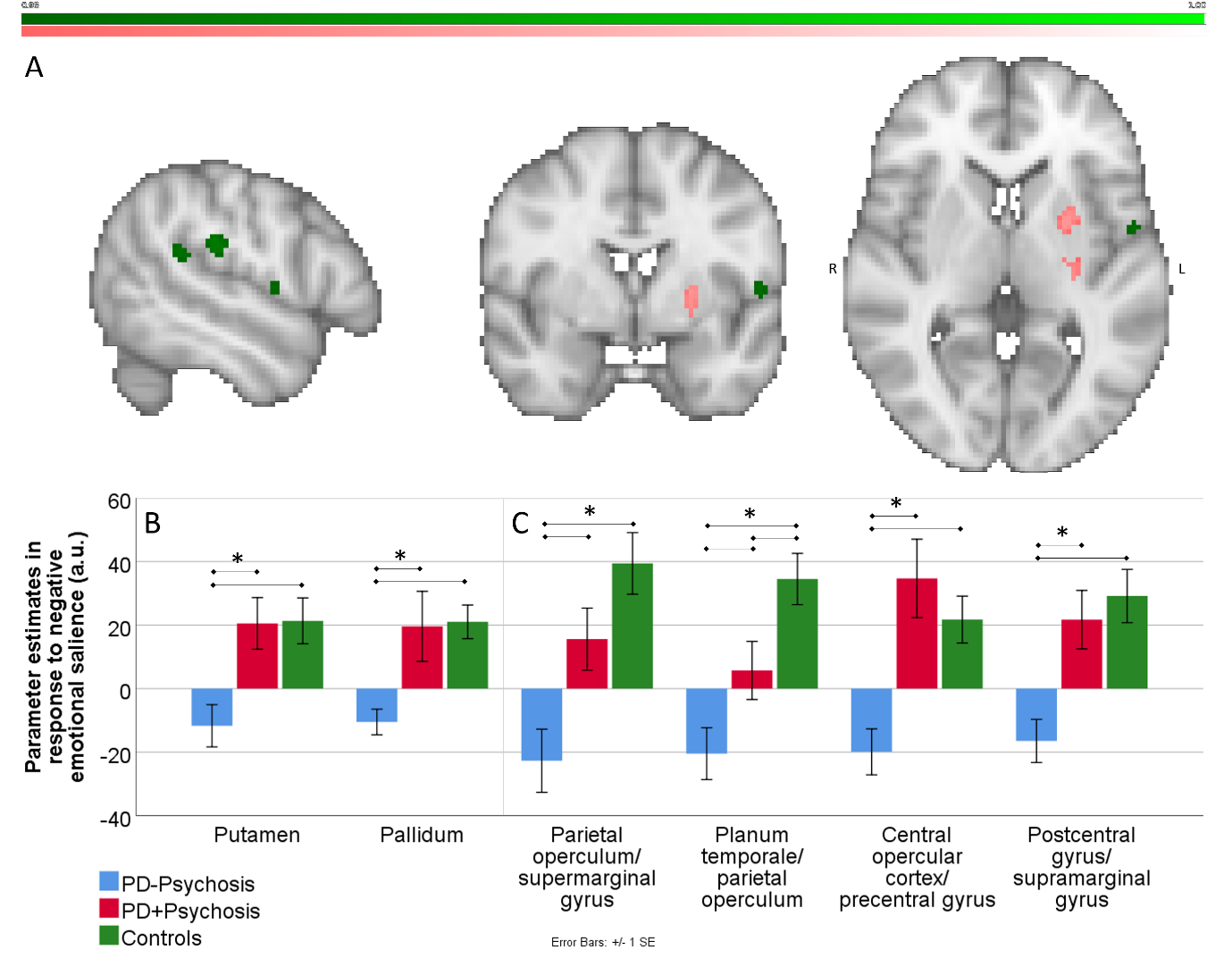


**Supplementary Figure 2.** Group effects for the voxelwise region of interests (ROI) analysis as well as whole brain analysis. A) Significant brain activations (slice location: x=-52, y=0, z=4) from the ROI analysis in putamen and pallidum (pink-to-white colour coding; maximal difference at x=0, y=-20, z=-6); and whole brain effects (dark green-to-light green colour coding) revealed four clusters including the pre- and postcentral gyrus and the supramarginal gyrus. Colour bars show significance level from p=0.05 (0.95) and lower (1). B/C) Bar charts showing extracted parameter estimates from significant clusters on the group level analysis as determined by the FSL randomised ANOVA, to visualise group differences. B) shows results of ROI analysis; C) results of whole brain analysis. Error bars show ±1 SE. PD-Psychosis: PD patients without psychosis, PD+Psychosis: PD patients with psychosis. L: left, R: right. *p<.05.

***fMRI activation to emotional salience: Multivariate analysis without controlling for covariates***

The multivariate test revealed a significant group effect on brain activation in response to negative emotional salience within the ROIs, controlling for resting CBF in these ROIs respectively, Pillai’s V=0.34, F(8,98)=2.47 p=.018. Tests of between-subject effects revealed significant group effects bilaterally in the amygdala, F(2,51)=5.57 p=.007, partial η^2^=.18, 83.5% power, hippocampus, F(2,51)=4.76 p=.013, partial η^2^=.11, 76.9% power as well as the striatum, F(2,51)= 3.26 p=.047, partial η^2^=.11, 59.5% power, and substantia nigra/VTA, F(2,51)= 3.62 p=.034, partial η^2^=.12, 64.4% power.

| **Supplementary Table 2:** Significant activations from group analysis (GLM) on Emotional – Neutral Oddball (Negative emotional salience) | | | | | | | |
| --- | --- | --- | --- | --- | --- | --- | --- |
| Anatomical structure | Hemisphere | Cluster size (voxel) | p-value | Peak Z score | Peak coordinates | | |
|  |  |  |  |  | x | y | z |
|  |  |  |  |  |  |  |  |
| ***Region of interest, incl. bilateral amygdala, bilateral hippocampus, dorsal and ventral striatum, and dopaminergic midbrain*** | | | | | | | |
| Putamen | L | 117 | 0.034 | 7.31 | -24 | 2 | 4 |
| Pallidum | L | 52 | 0.036 | 8.88 | -24 | -16 | 0 |
| ***Whole brain analysis*** | | | | | | | |
| Parietal operculum/supramarginal gyrus | L | 189 | 0.043 | 10.98 | -52 | -26 | 24 |
| Planum temporale/parietal operculum | L | 60 | 0.044 | 11.91 | -48 | -40 | 18 |
| Central opercular cortex/precentral gyrus | L | 22 | 0.047 | 12.2 | -54 | 0 | 6 |
| Postcentral gyrus/supramarginal gyrus | L | 19 | 0.049 | 10.44 | -42 | -26 | 40 |
| FSL randomise, 5000 permutation, FWE cluster-corrected results. | | | | | | | |


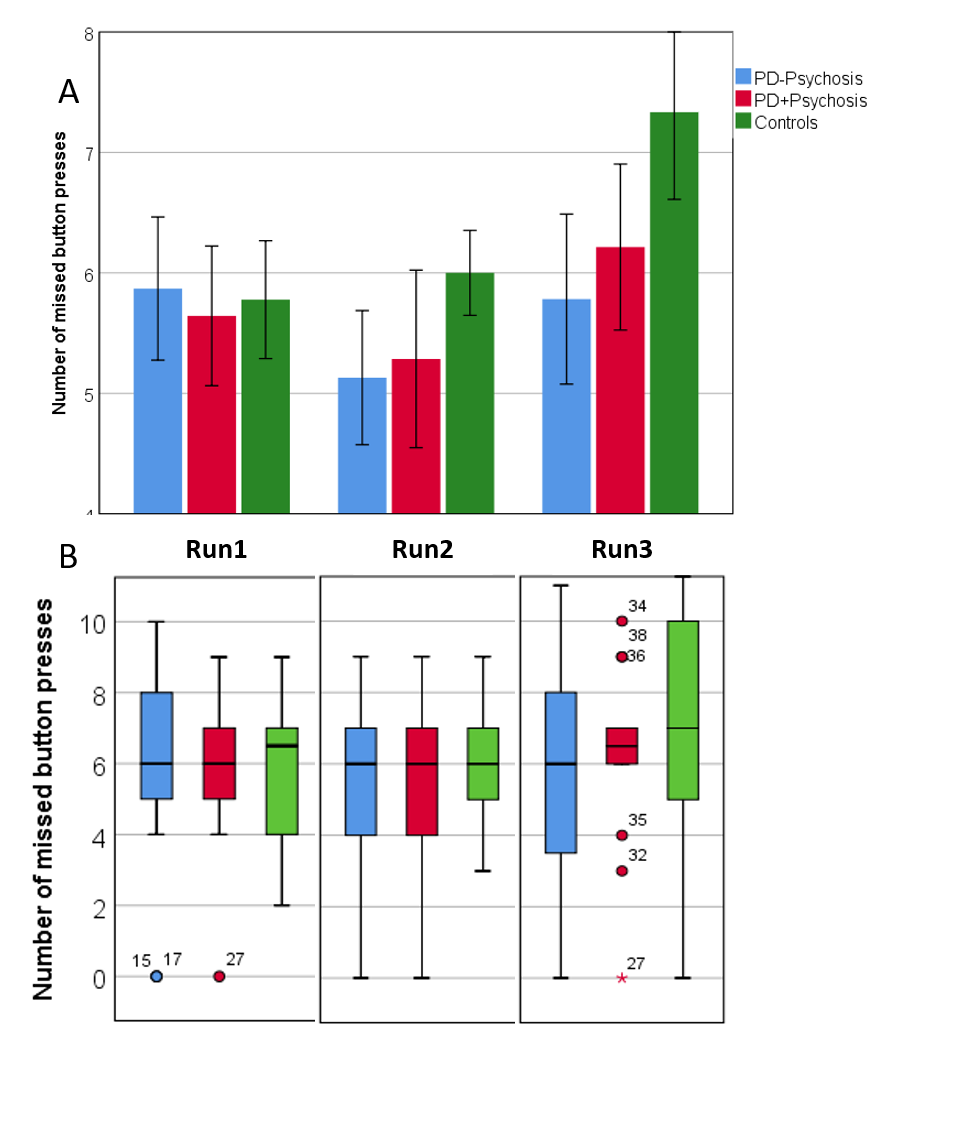


**Supplementary Figure 3 A.** Bar chart shows number of missed button presses across groups and testing blocks (error bar ± 1 SE). **B.** Box plot shows number of missed button presses across groups and testing blocks, by showing the minimum, first quartile, median, third quartile, and maximum.


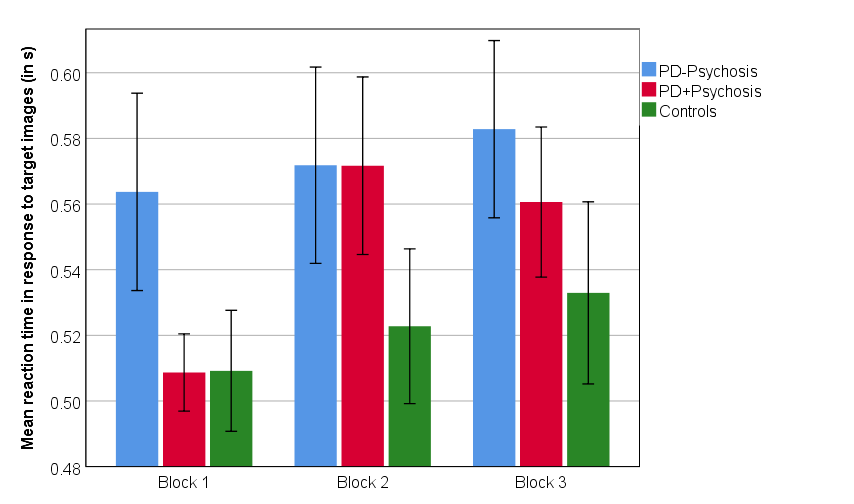


**Supplementary Figure 4.** Bar chart shows mean reaction times across groups and testing blocks (error bar ± 1 SE).


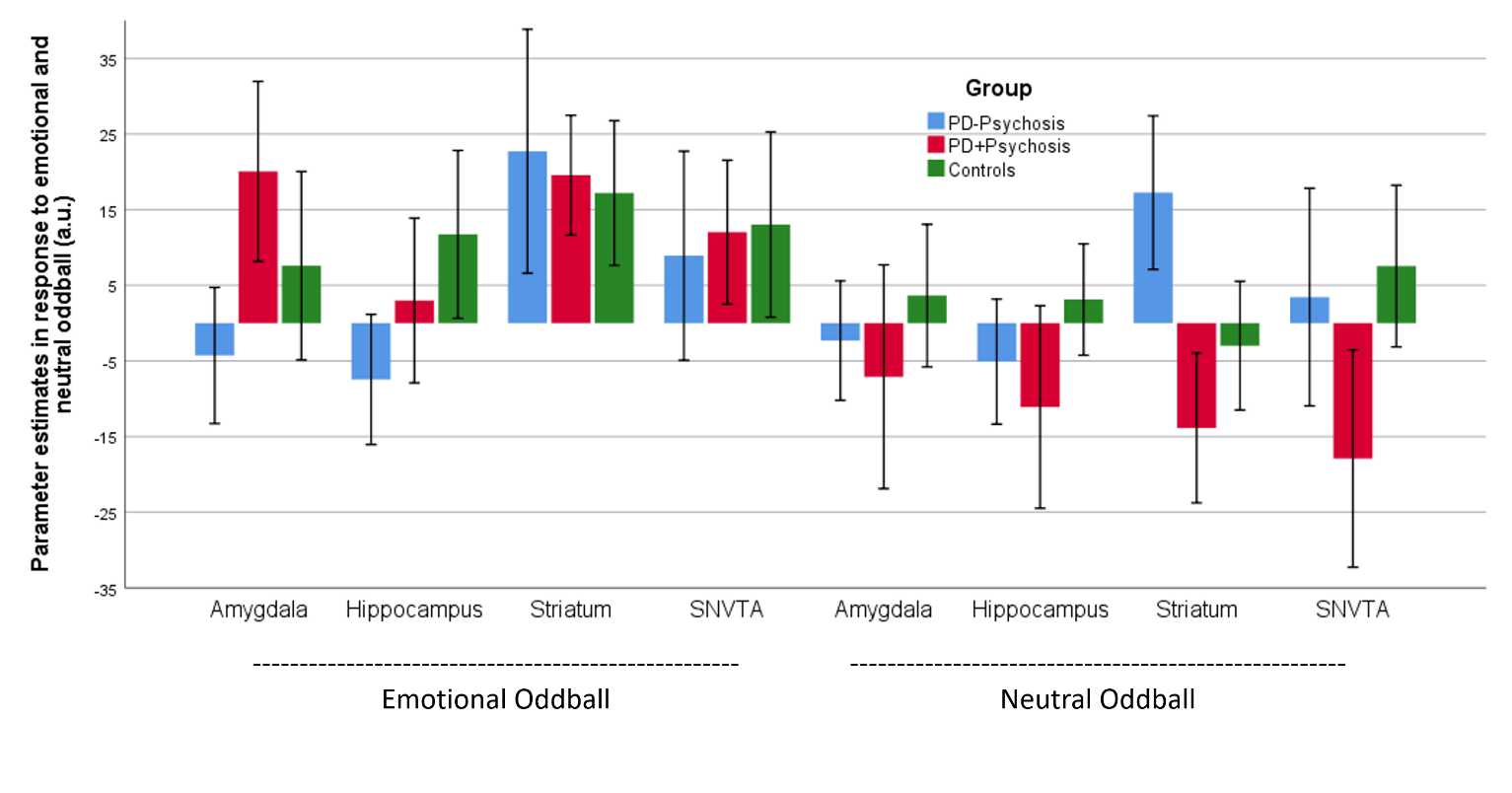


**Supplementary Figure 5.** Parameter estimates in response to emotional and neutral oddball samples separately. The parameter estimates indicate the potential drivers of the COPE (contrast of parameter estimates) effect (error bar ± 1 SE).

***‘Jumping to conclusion’ task in a reduced sample: probabilistic reasoning***

Additionally, some participants completed a ‘jumping to conclusion’ task (Ermakova et al., 2019): 12 of 23 PD patients without psychosis, 5 of 15 patients with psychotic symptoms, and 18 of 19 controls subjects. Due to the incomplete nature of the data we treat these data as preliminary results.

*Task description*

In order to test a possibly impaired top-down decision-making ability, we tested the ‘jumping to conclusion’ behaviour in the participants of this study. The task is described in detail in (Ermakova et al., 2019). Participants were told that two lakes, a golden and a black lake, contain golden and black fish with a ratio of 40:60 and 60:40 respectively. Participants had to draw out a sequence of fish and were then asked to make a decision about the specific lake the fish were coming from. The task consisted of four blocks of increasing difficulty: In block 1 participants had to guess whether the fish were drawn from the golden or the black lake after however many fish they drew out and having received feedback as to whether their choice was correct. In block 2 a reward for correct guessing was introduced (100 points) and a loss (-100 points) for wrong guessing. In blocks 1 and 2 participants could draw up to 20 fish. In block 3, a cost for every extra fish after the first one was introduced and 5 points were deducted from the possible total win of 100 points. Block 4 was similar to block 3, however the information sampling cost increased incrementally (the first fish scored 0 points, then -5 points, then -10, -15 etc.). The more fish that were sampled, the more points were lost. Our measures of interest were ‘draws to decision’ (DTD) and probability of correct decisions (probability correct).

*Description of the reduced sample*

Unfortunately, the data set was incomplete; we were successful in collecting data from 12 of 23 PD patients without psychosis, 5 of 15 patients with psychotic symptoms, and 18 of 19 control subjects. Those participants who did not continue with the testing, reported tiredness after the scan and interview, and wished not to continue with the behavioural testing. For the two patient groups we compared the subgroup who completed the ‘jumping to conclusions’ task to the subgroup who did not complete that part of the study. We did not find differences in the two subgroups of the PD patients without symptoms in terms of demographics or symptom scores apart from the GAF disability score which was significantly higher in the group that completed the ‘jumping to conclusions’ task (F(1,23)=4.28, p=.041). The subgroups of the PD patients with psychotic symptoms also did not differ on demographics, but were significantly different on GAF (F(1,13)=5.89, p=.032), which was again higher for those who completed the task, and on BDI, which was significantly lower for those who completed the task.

***‘Jumping to conclusion’ analysis in a reduced sample***

We applied a repeated measure ANOVA across the four blocks controlling for LED and Mini-Mental-State Examination (MMSE) score. We ran one repeated measure analysis for DTDs and one for probability correct.

***‘Jumping to conclusion’ results in a reduced sample***

Exploring DTD, we did not find a significant group effect (F(2,30)=2.725, p=.082, 50% power), controlling for MMSE (F(1,30)=7.556, p=.01, 75.8%power). See Supplementary Figure 4A for a boxplot presentation of DTD across all blocks.

For the probability of making a correct choice (probability correct), we found a significant group effect (F(2,30)=4.617, p=.020, 72.2%power), and a significant covariate effect for MMSE (F(1,30)=7.692, p=.019, 66.9% power). We explored the group effect in a *post hoc* analysis and found that PD patients with psychotic symptoms had a significantly lower probability of correctly identifying the correct lake than PD patients without psychotic symptoms (p=.006) and controls (p=.043; mean probability correct: PD with psychotic symptoms: .57 (SE.03), PD without psychotic symptoms: .69 (SE.02), controls: .664 (SE.02). See Supplementry Figure 4B for a boxplot presentation of the probability of being correct across all blocks.


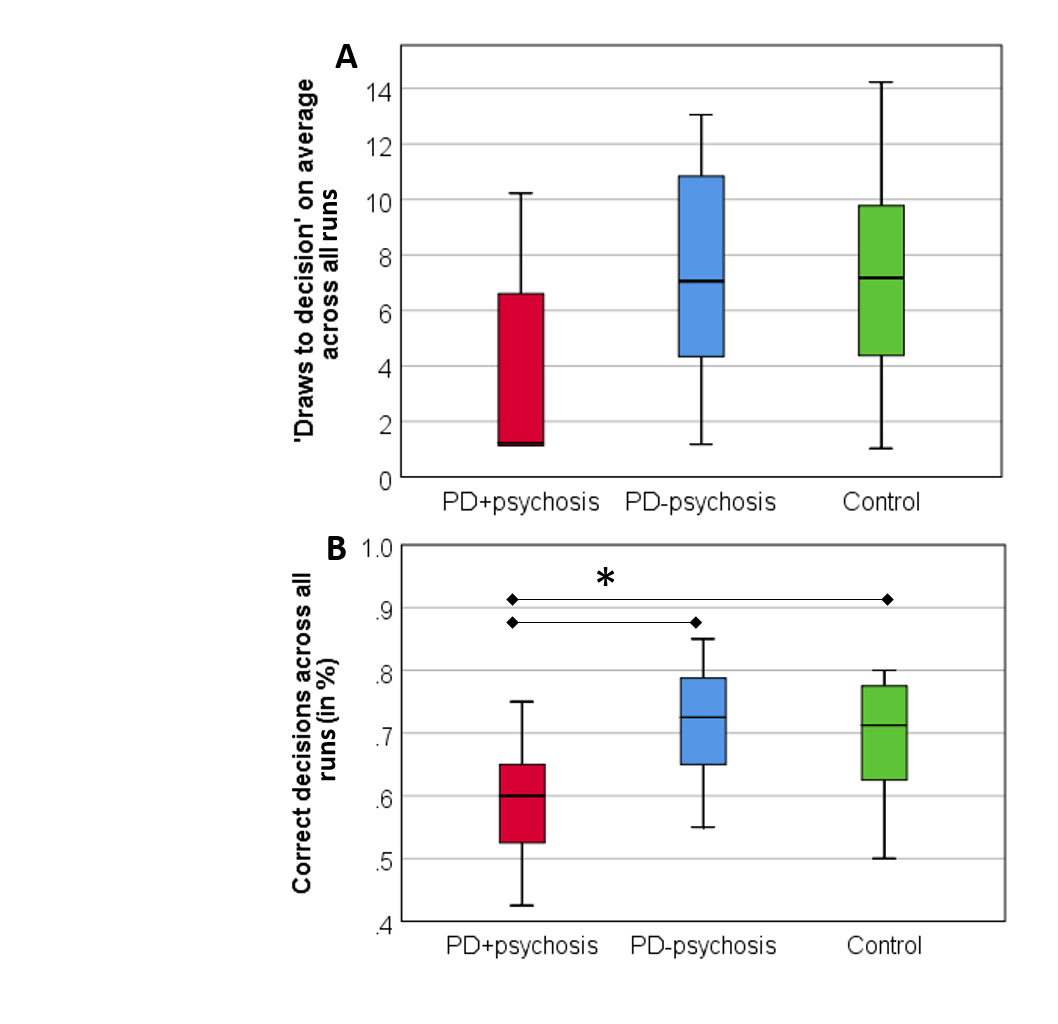


**Supplementary Figure 6.** Box plot shows results of ‘jumping to conclusions’ task across all blocks. A) shows the number of draws, participants take to make a decision. B) shows the percentage of correct decisions. The box plot shows the minimum, first quartile, median, third quartile, and maximum of each group.
